# In vitro to in vivo extrapolation to derive a metabolism factor for estimating the aggregate exposure to salicylic acid after dermal exposure of its esters

**DOI:** 10.1007/s00204-024-03749-8

**Published:** 2024-04-24

**Authors:** Abdulkarim Najjar, Sebastien Grégoire, Beate Nicol, Andreas Natsch, Nazanin Golbamaki, Fanny Boisleve, Amaia Irizar, Brian Wall, Angus Swinscoe, Valérie Masini-Etévé, Dan Selechnik, Anne Marie Api, Peter Griem, Nicola Hewitt, Estefania Cardamone

**Affiliations:** 1 Beiersdorf AG, Beiersdorfstr. 1-9, 20245 Hamburg, Germany; 2 L’Oreal Research & Innovation, Aulnay-Sous-Bois, France; 3 Safety and Environmental Assurance Centre, Unilever UK, Colworth Science Park, MK44 1LQ Sharnbrook, United Kingdom; 4 Givaudan Schweiz AG, CH-8310 Kemptthal, Switzerland; 5 Chanel, Neuilly, France; 6 The International Fragrance Association (IFRA), Geneva, Switzerland; 7 Colgate-Palmolive Company, Piscataway, NJ 08854 USA; 8 Whitman Laboratories, The Estée Lauder Companies, Petersfield, United Kingdom; 9 L’Oréal Recherche & Innovation, Clichy, France; 10 Research Institute for Fragrance Materials (RIFM), Inc., Woodcliff Lake, NJ USA; 11 Symrise AG, Holzminden, Germany; 12 SWS, Erzhausen, Germany; 13 Cosmetics Europe, Brussels, Belgium

**Keywords:** IVIVE, Salicylate esters, Salicylic acid, Metabolism factor, Safety assessment, High-throughput pharmacokinetic modelling

## Abstract

As part of the safety assessment of salicylate esters in cosmetics, we developed a metabolism factor based on in vitro to in vivo extrapolation (IVIVE) to provide a better estimation of the aggregate internal exposure to the common metabolite, salicylic acid. Optimal incubation conditions using human liver S9 were identified before measuring salicylic acid formation from 31 substances. Four control substances, not defined as salicylic esters but which could be mistaken as such due to their nomenclature, did not form salicylic acid. For the remaining substances, higher in vitro intrinsic clearance (CL_int, in vitro_) values generally correlated with lower LogP values. A “High-Throughput Pharmacokinetic” (HTPK) model was used to extrapolate CL_int, in vitro_ values to human in vivo clearance and half-lives. The latter were used to calculate the percentage of substance metabolised to salicylic acid in 24 h in vivo following human exposure to the ester, i.e. the “metabolism factor”. The IVIVE model correctly reproduced the observed elimination rate of 3 substances using in silico or in vitro input parameters. For other substances, in silico only-based predictions generally resulted in lower metabolism factors than when in vitro values for plasma binding and liver S9 CL_int, in vitro_ were used. Therefore, in vitro data input provides the more conservative metabolism factors compared to those derived using on in silico input. In conclusion, these results indicate that not all substances contribute equally (or at all) to the systemic exposure to salicylic acid. Therefore, we propose a realistic metabolism correction factor by which the potential contribution of salicylate esters to the aggregate consumer exposure to salicylic acid from cosmetic use can be estimated.

**Supplementary Information:**

The online version contains supplementary material available at 10.1007/s00204-024-03749-8.

## Introduction

The safety assessment of cosmetic ingredients requires a reliable and quantitative estimate of exposure. For topically applied cosmetic ingredients, the systemic exposure is adjusted according to the skin absorption of the substance. Exposure assessments have been conducted to evaluate the safety of a single ingredient that is present in multiple product types, e.g., parabens (Ouedraogo et al. 2022), or in multiple product types and in the diet, e.g., vitamin A (Tozer et al. 2019) and aluminium (SCCS 2022a). Notably, the prediction of the systemic aggregate exposure to aluminium considers multiple aluminium compounds and their equivalent stoichiometric ratios of aluminium. The use of the molecular weight to convert to the substance of interest, together with the dermal penetration of the source substance, have been used to assess the safety of aggregate exposures (SCCS 2022a, 2022b). Another scenario which can potentially necessitate an aggregated exposure estimate of a single substance is when different chemicals produce a common metabolite which is considered to represent a toxicological concern. This scenario is relevant to the potential systemic exposure to salicylic acid as a metabolite from the use of certain esters of salicylic acid (referred to from here as “salicylate esters”) as ingredients in cosmetic products. The current uses of salicylic acid itself are safe for use in cosmetics (SCCS 2019, 2022b). Since there are several salicylic esters, as well as salicylic acid, that can be present in cosmetic products, there is a potential for additive systemic exposure of consumers to salicylic acid (SCCS 2020, 2021).

**Fig. 1:**
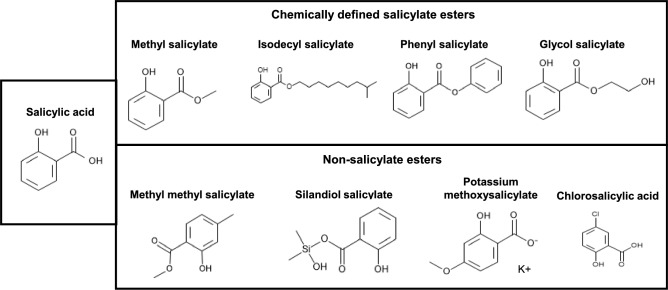
Structure of salicylic acid and examples of some of the substances tested. These include substances chemically defined as salicylate esters and four which are not

As part of the exposure estimate to salicylic acid due to the use of salicylate esters in different cosmetic products and, at times, the use of more than one salicylate ester in a given product, the systemic exposure dose (SED) can be corrected according to the relative molecular weight of the parent ester and salicylic acid, where for most substances, one mole of salicylate ester is stoichiometrically equivalent to one mole of salicylic acid. A second correction factor is the skin absorption of the parent ester, whereby lower skin absorption is linked to lower SED. However, we propose a third correction factor, namely a “metabolism factor”, since not all salicylate esters would be expected to release the same amount of salicylic acid over a given time frame (as observed for homosalate, which is not 100% hydrolyzed after topical application at the maximal dose in human volunteers (Matta et al. 2020)). As such, they cannot be regarded as equal in terms of their contribution to systemic concentrations of salicylic acid. Therefore, we employed in vitro and in silico methods to help derive metabolism factors for salicylate esters for use in a probabilistic exposure estimate. The substances tested were selected from the complete list of substances present in three databases (Cosing (https://single-market-economy.ec.europa.eu/sectors/cosmetics/cosmetic-ingredient-database_en), ECHA’s Assessment of Regulatory Needs of Salicylate Esters (ECHA 2021) and the IFRA transparency list (https://ifrafragrance.org/priorities/ingredients/ifra-transparency-list)). This list of substances is qualitative and independent of the product type used or the current tonnage in the EU. The structure of salicylic acid and examples of some of the salicylate esters are shown in Fig. 1 (the structures of the complete list of salicylate esters tested are shown in Supplementary Table 1). The Cosing database also contains several ingredients that might appear to be salicylic esters due to their nomenclature but are not, by chemical definition, esters of salicylic acid. Therefore, four of these were included in the study as controls which are not expected to form salicylic acid (methyl 4-methylsalicylate, silandiol salicylate, potassium methoxysalicylate, and chlorosalicylic acid (Fig. 1)). The experiments measured the formation of salicylic acid in incubations of a total of 31 test substances with human liver S9 to determine whether the selected substances formed salicylic acid and, if so, the rate at which it was formed.

It could be assumed that many of the salicylate esters could be completely metabolised in the body, which would be considered a worst-case default assumption for the safety assessment. However, while these substances are metabolised in in vitro incubations, the extent of metabolism over 24 h in vivo may not be 100%. For example, homosalate is completely converted to salicylic acid under in vitro conditions by human liver microsomes in 20 min (SCCS 2020) but in vivo, only 19–30% is converted by humans over 24 h (based on the terminal half-life of homosalate in plasma reported by Liu et al. (2020) (see calculation for this in the Methods section)). This means that metabolism data from in vitro incubations (which are purposely designed to achieve a good sensitivity for detecting metabolites) need to be translated into in vivo relevant rates, i.e. an in vitro to in vivo extrapolation (IVIVE) needs to be conducted. An IVIVE was defined by Najjar et al. (2022) as “extrapolating kinetic parameters measured in vitro to estimates of the associated kinetics in vivo”, which is essential in simulating the fate of a substance in the human body. Therefore, we used a “High-Throughput Pharmacokinetic” (HTPK) Model from SimulationPlus (https://www.simulationsplus.com/software/admetpredictor/high-throughput-pharmacokinetic-simulation/), described by Liu et al. (2020) and Naga et al. (2022), to convert in silico and/or in vitro derived intrinsic clearance values (CL_int, in vitro_) to in vivo clearance and half-life values. This high-throughput model was chosen for IVIVE in this study since it allowed the simulations for multiple substances to be conducted in a relatively short time, while providing good confidence in the resulting values by comparing predicted values with measured in vivo values (conducted herein). This approach is also adopted by pharmaceutical companies as part of their screening process and by researchers conducting safety evaluations of non-pharmaceutical chemicals with environmental relevance (Naga et al. 2022; Wambaugh et al. 2018). The predicted in vivo human half-life was then used to estimate the percentage of the substance metabolised to salicylic acid in 24 h in vivo—referred to here as the so-called “metabolism factor”. A value of 0 for substances which are not converted to salicylic acid at all would mean the systemic exposure is zero because no salicylic acid is formed in vivo. On the other hand, substances which are converted to salicylic acid will have a higher factor, e.g., substances that are completely converted to salicylic acid within a day will have a factor of 100%.

We describe here how the in vitro incubations were optimised, especially considering the highly lipophilic nature of some of the salicylate esters, and the use of the resulting CL_int, in vitro_ values as input for the HTPK model. The impact of the use of in silico versus in vitro parameters on the metabolism factors is also described.

## Methods

### Selection of substances

In a first step all mixtures and substances with an INCI or substance name indicating the substance is a salicylate ester were collected. The INCI and CAS numbers were confirmed and the structures, e.g., ester of salicylic acid, diester, mixture, analogues of salicylic acid, etc., were assessed by chemists from four partners and a consolidated list was completed. In a final step, the inclusion of the substances was discussed. These were restricted to single substances which were classified as esters of salicylic acid according to the structure shown in Fig. 1.

Substances that might appear to be salicylic esters due to their nomenclature but are not, by chemical definition, esters of salicylic acid were tested as controls (methyl 4-methylsalicylate, silandiol salicylate, potassium methoxysalicylate, and chlorosalicylic acid (Fig. 1).

### Chemicals and reagents

All chemicals were of the highest purity available and were either purchased from Sigma-Aldrich (Germany), TH Geyer (Germany) or Lolab (Germany) or were provided from in-house supplies. Human liver S9 fractions (mixed gender, 200-donor-pool, Catalogue number H2610.S9, lot 110,370) were from Xenotech. Bovine serum albumin (Catalogue number BSA03117332001, Lot 10,078,322) fraction V was from Roche.

### Incubations with human liver S9

All stock and working solutions were prepared in glass vials to minimise potential non-specific binding. Test substances and the reference chemical, 7-ethoxycoumarin, were prepared as 10 mM stock solutions in DMSO or acetonitrile, respectively. The stock solutions were further diluted in the respective solvent to 600 µM start solutions. The final test concentration in the assay was 5 µM in presence of 0.8% DMSO for test substances or 0.8% acetonitrile for 7-ethoxycoumarin. Assays were performed in 48-well glass-coated WebSeal™ Plate + PP microtiter plates from Thermo Fisher Scientific. Experiments were conducted on a horizontal shaker with a fitted heating block (“Thermomixer”, Eppendorf). The incubations (total volume 1100 µL/well) were in triplicate and consisted of 0.1 mg/mL human liver S9 fractions in sodium phosphate buffer (0.1 M, pH 7.4, 5 mM MgCl_2_, 0.01% pluronic acid, and 0%, 1% or 4% bovine serum albumin (BSA)) supplemented with cofactors (150 µM NADPH and 150 µM NAD) and test substance. All solutions and plates were pre-incubated for 10 min at 37 °C before starting the incubation. The assay buffer, supplemented with the cofactors (1070 µL) and 10 µL of the test substance start solution, were pipetted into the respective wells of the incubation plate and incubated for 15 min at 37 °C and mixed by rotating the plate at 450 rpm. At time point 0 min, an aliquot of 90 µL was removed from the wells, transferred to a separate glass-coated plate and pre-quenched with 200 µL stop solution (100% acetonitrile containing the internal standards, 1 µM diazepam, 1 µM griseofulvin, 10 µM diclofenac, and 1 µM warfarin) before mixing with 10 µL S9 fractions (1 mg/mL). For all other time points, the incubation was initiated by addition of 110 µL S9 fractions (1 mg protein/mL) per well, yielding a final protein concentration of 0.1 mg/mL in the incubations. Serial sampling was performed at sampling time points 5, 15, 30, 120 and 240 min. Aliquots of 100 µL were removed from the incubations and quenched with 200 µL stop solution containing the ISTDs.

Negative controls containing the test chemicals and cofactors but without S9 fractions were run in parallel to each assay in triplicate. Sampling time points for the negative control were 0, 15 and 240 min. Reference controls, 7-ethoxycoumarin (in acetonitrile) and benzyl salicylate (in DMSO), were tested in parallel to each assay in duplicates. Samples were taken after 0, 30 and 120 or 240 min.

### Sample preparation and LC–MS analysis

A volume of 200 µL acetonitrile containing the internal standards was added to 100 µL of test, reference or calibration standard sample. After mixing and centrifugation (2200 g, 5 min), the supernatants were transferred to appropriate glass vials and subjected without further dilution to liquid chromatography–mass spectrometry (LC–MS) analysis.

Samples were analysed for the presence of salicylic acid by LC–MS or liquid chromatography–high resolution mass spectrometry (LC–HRMS). The analytes were separated on a HPH Poroshell C18-analytical column 2.7 µm, 100 × 3.0 mm (Phenomenex, Germany) with a corresponding pre-column C6-Phenyl, 4 × 2.0 mm using the gradients shown in Supplementary Table 2. Detection of salicylic acid was performed using the Orbitrap™ technology with accurate mass. The scan range, mass resolution and detection mode are listed in Supplementary Table 3. The accurate mass of the monitoring ions ± 10 mDa were used for test item peak integration. The LC–MS equipment comprised a Surveyor Plus (Thermo Fisher Scientific, USA) HPLC system connected to a TSQ Quantum Discovery Max (Thermo Fisher Scientific, USA) triple quadrupole mass spectrometer and equipped with an electrospray (ESI) or APCI interface (Thermo Fisher Scientific, USA) connected to a PC running the standard software Xcalibur 2.0.7. The LC–HRMS: HPLC system comprised a Vanquish Quaternary pump, a Vanquish column compartment and a Vanquish split sampler (Thermo Fisher Scientific, USA) connected to a Q-Exactive (Orbitrap) or a Q-Exactive Plus mass spectrometer (Thermo Fisher Scientific, USA). Data handling was performed with the standard software Chromeleon 7.2 SR5 MUf. The injection volume was 3 µL and the pump flow rate was 600 µL/min.

For 7-ethoxycoumarin, the pump flow rate was 600 µL/min and the analytes were separated on a Kinetex Phenyl-Hexyl analytical column 2.6 µm, 50 × 2.0 mm (Phenomenex, Germany) with a corresponding pre-column using the gradients as presented in Supplementary Table 2. Detection of 7-ethoxycoumarin was performed applying the triple quadrupole technology. Ions with the highest signal-to-noise ratio were used to quantify 7-ethoxycoumarin in the selected reaction monitoring mode and as qualifier, respectively. The scan range, mass resolution and detection mode are indicated in Supplementary Table 3.

The concentrations of salicylic acid from all samples from incubations with test substances were quantified using a calibration curve with 6 concentrations in duplicate between 25 nM and 10 µM for salicylic acid. For reference control samples, a single concentration of 5 µM for 7-ethoxycoumarin was used. All standards were prepared in the standard matrix corresponding to assay conditions (i.e. of 0.1 mg/mL human liver S9 fractions in phosphate buffer (see above)). Specificity was controlled with blanks of acetonitrile only and acetonitrile in standard matrix. The accuracy of the salicylic acid calibration points was within 100 ± 15%, except for one replicate at 200 nM. The lowest limit of quantification (LLOQ) was defined as the lowest standard concentration used for the corresponding calibration curve and was 50 nM. All calibration curves were validated according to the guidelines for bioanalysis (FDA 2018).

### Data analysis

The in vitro intrinsic clearance values (CL_int, in vitro_) were calculated based on the concentration of salicylic acid formed. The amount of parent substance was calculated by subtracting the concentration of salicylic acid formed (in µM) from the initial nominal concentration (which was 5 µM in all incubations). The CL_int, in vitro_ was calculated by first determining the in vitro half-life (*t*_1/2_) from the slope (*k*) of the linear regression of the depletion of the parent compound, i.e. natural logarithm, ln, of the % remaining versus incubation time (Eq. 1):

1 $${t}_{1/2} = \frac{- 0.693}{k}.$$

The t_1/2_ was then used to calculate the CL_int, in vitro_, expressed as µL/min/mg S9 protein, according to Eq. 2:

2 $${\text{CLint}},\;invitro = \frac{{(V \times 0.693)}}{{t_{{1/2}} }},$$

where V is $\frac{1}{{0.1\;mg/mLS9\;protein}}$ = 10,000 µL/mg protein.

Two kinds of the in vitro intrinsic clearance were obtained as a result: “Unrestricted CL_int, in vitro_*”* refers to the clearance without the incorporation of the unbound fraction (derived from the incubation with liver S9 without BSA), whereas “restricted CL_int, in vitro_*”* refers to the clearance of the free fraction (i.e. in the presence of BSA).

### HTPK model simulations

The High-Throughput PharmacoKinetics (HTPK) model from SimulationPlus (https://www.simulationsplus.com/software/admetpredictor/high-throughput-pharmacokinetic-simulation/), described by Liu et al. (2020) and Naga et al. (2022) was used to estimate the t_1/2_ in human plasma. It is a simplified physiologically based pharmacokinetic (PBPK) model, which includes stomach, intestinal, liver, and central (general circulation and other organs) compartments. As described by Naga et al. (2022), the central compartment volume was set so that it is equal to the steady-state volume of distribution (*V*_ss_) estimated using the Rodgers and Rowland method, as modified by Lukacova et al. (Lukacova et al. 2008).

For all simulations: No renal clearance of the parent substance was considered. The calculation estimates the terminal *t*_1/2_, when distribution is no longer applicable and only the elimination of the parent is followed. It was assumed (conservatively) that the metabolism to salicylic acid is the only route of the parent substance elimination. On the other hand, the converted amount of salicylic acid is rapidly eliminated from the body (Burke et al. 2005); therefore, there is no accumulation.

Model inputs were: Physiological parameters (default values for the HTPK model (Naga et al. 2022; SimulationsPlus website): human weight = 70 kg; liver weight = 1433 g; liver blood flow = 87 L/h; chemical-specific (relevant for the study): physicochemical properties; volume of distribution (V_d_) and CL_int, in vitro_. The restricted CL_int, in vitro_ (expressed as µL/min/mg S9 protein) was scaled up to in vivo restricted intrinsic clearance (CL_int,in vivo_, expressed as µL/min/kg body weight (BW)) using Eq. 3:

3 $$CL_{{int,invivo}} = CL_{{int,invitro}} \times mgS9\frac{{protein}}{g}\;liver \times g\frac{{liver}}{{kg}}BW,$$

where the scaling factor for S9 is 107.3 mg S9 protein/g liver (Wang et al. 2020) and the liver weight is 1433 g for 70 kg body weight (BW) (default values for the SimulationsPlus HTPK software).

To calculate the hepatic clearance, the HTPK Model uses values for the fraction unbound in the plasma (*F*_up_), the blood-to-plasma ratio (*R*_bp_), the blood flow to the liver (*Q*_H_) and the unrestricted in vivo intrinsic clearance (CL_int,in vivo_), according to the well-stirred model represented Eq. 4:

4 $${CL}_{H}\left(\frac{L}{h}\right)=QH. \frac{{R}_{bp} . \frac{{F}_{up}}{{F}_{inc}} . {CL}_{int,in vivo}}{{Q}_{H} . {R}_{bp}+ \frac{{F}_{up}}{{F}_{inc}}. {CL}_{int,in vivo}}.$$

The F_up_ and R_bp_ were predicted using the ADMET Predictor software from SimulationsPlus (predicted values are summarised in Supplementary Table 1). The binding of the substances in the incubation with liver S9 plus 4% BSA was assumed to be the same as that occurring in human plasma (Haab et al. 2005) (i.e. F_up_/F_inc_ in Eq. 4 is equal to 1).

The calculations used values of restricted CL_int,in vitro_ from measured values in liver S9 and using predicted values from HTPK Model in SimulationsPlus. Of note, the HTPK Model does not incorporate intrinsic clearance values from liver S9 but from liver microsomes. Therefore, an adjustment factor was derived to calculate the equivalent in vivo hepatic clearance for microsomes by dividing the scaling factor (SF) for liver S9 by the SF for microsomes:

$${\text{Adjustment}}\;{\text{factor}} = \frac{{S9SF}}{{{\text{Microsome}}\;SF}} = \frac{{107.3\;mg\;S9{\text{protein}}/{\text{gliver}}}}{{38\;{\text{mg}}\;{\text{microsomal}}\;{\text{protein}}/{\text{gliver}}}} = 2.84.$$

Therefore, the values of scaled in vivo hepatic clearance measured in liver S9 were multiplied by 2.84 and used as input to the HTPK Model. This model calculates the kinetics of the substance and values such as the volume of distribution (Liu et al. 2020; SimulationsPlus website). The output of the HTPK in these simulations was a t_1/2_ in humans, which was used to calculate the metabolism factor, i.e. % of the conversion of the substance, according to Eq. 5:

5 $${\text{Metabolism}}\;{\text{Factor}}\left( \% \right) = \left( {1 - 0.5^{{\left( {\frac{t}{{t_{{half - life}} }}} \right)}} } \right) \times 100,$$

where *t* is the time passed since exposure of the chemical; 24 h (1 day) and in vivo *t*_1/2_ is the half-life of a substance in humans.

## Results

### In vitro incubations—optimisation and impact of BSA

**Fig. 2:**
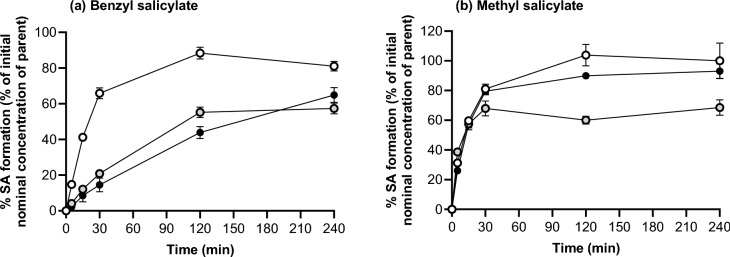
Formation of salicylic acid in incubations of A benzyl salicylate and B methyl salicylate with human liver S9 supplemented with 0% (open symbols), 1% (grey symbols) and 4% (black symbols) (w/v) BSA. Values are a mean of triplicate samples ± SD

In incubations conducted in the absence of BSA, 90–100% of the nominal concentrations of benzyl salicylate and methyl salicylate were converted to salicylic acid (Fig. 2), indicating that loss of parent substance is directly proportional to the formation of salicylic acid. The initial formation of salicylic acid from benzyl salicylate was reduced by the inclusion of 1% and 4% BSA in the incubation, whereby the % salicylic acid formed by 15 min was 59.6 ± 0.9%, 20.9 ± 1.8% and 14.5 ± 3.9% of the nominal dose in incubations with 0%, 1% and 4% BSA, respectively. By contrast, the presence of BSA did not markedly impact the initial formation of salicylic acid from methyl salicylate (the % salicylic acid formation after 15 min was 59.6 ± 3.3%, 57.5 ± 2.7% and 57.1 ± 3.6% of the nominal dose in incubations with 0%, 1% and 4% BSA, respectively). A concentration of 4% BSA was tested since this is the reported concentration of BSA in the plasma (Haab et al. 2005). A concentration of 1% BSA was included to determine how the BSA concentration affected the CLint and CLH. When the *t*_1/2_ values from the incubations were scaled to in vivo hepatic clearance (CL_H_), the values were the same regardless of the concentration of BSA used in the incubations (Table 1), where the liver blood flow (*Q*) is the rate-limiting step for metabolism. Salicylic acid was not metabolised in incubations without or with BSA (there was no depletion over 4 h (data not shown)).

**Table 1:** Conversion of in vitro half-lives to in vivo hepatic clearance values

Substance	BSA concentration (% w/v)	Half-life (min)	CL_int in vitro_ (µL/min/mg S9 protein)	CL_int in vivo_ (L/h)	CL_H_ (L/h)
Methyl salicylate	0	13.1 ± 1.4	535 ± 59	4935 ± 542	83.3 ± 0.4
	1^a^	20.0 ± 2.9	351 ± 50	3514 ± 676	84.8 ± 0.4
	4^a^	13.6 ± 0.9	512 ± 36	4720 ± 330	85.4 ± 0.1
Benzyl salicylate	0	19.1 ± 1.9	365 ± 38	3368 ± 355	83.7 ± 0.3
	1^a^	103.6 ± 13.6	68 ± 9	624 ± 81	76.3 ± 1.2
	4^a^	165.0 ± 20.2	42 ± 5	392 ± 49	71.1 ± 1.6

The half-life was scaled up to in vivo hepatic clearance using Eq. 4. Measured values of *F*_up_ were used for methyl salicylate (ECHA 2019)) and benzyl salicylate (unpublished Cosmetics Europe Long Range Science Strategy (LRSS) data). ^a^The free fraction of substance in vivo in plasma was assumed to be the same as that in incubations with BSA (therefore, *F*_up_ was not included in Eq. 4). Binding to the S9 proteins was considered to be negligible compared to that to BSA; therefore, no additional adjustments were made based on liver S9

### In vitro clearance of test substances

Based on these results of the pilot study, the design of the main experiments testing 31 substances included 4% BSA to represent in vivo plasma binding and reduce non-specific binding and increase the solubility of the more lipophilic substances.

### Substances minimally metabolised to salicylic acid

Under the optimised conditions, two substances were not metabolised to salicylic acid at all (potassium methoxysalicylate and silandiol salicylate) and two substances from which only trace amounts were detected after 2 h and 4 h incubation with liver S9 (methyl 4-methylsalicylate and chlorosalicylic acid—no salicylic acid was formed in the control incubations without S9) (Table 2). These substances were not expected to be hydrolysed to salicylic acid, but were included in these experiments for completeness. Furthermore, these examples could help to support the grouping of substances according to whether or not they contribute to systemic salicylic acid exposure. While two substances did form salicylic acid, the concentrations formed were ~ 100-fold lower than that formed by the positive control, benzyl salicylate, and were therefore not investigated further.

**Table 2:** Results for substances not classified as salicylate esters incubated with liver S9

Substance	Concentration of salicylic acid present in the incubation (nM)		
	0.5 h	2 h	4 h
Potassium methoxysalicylate	0 ± 0	0 ± 0	0 ± 0
Silandiol salicylate	0 ± 0	0 ± 0	0 ± 0
Methyl methyl salicylate	0 ± 0	17.9 ± 9.4	38.2 ± 10.4
Chlorosalicylic acid	0 ± 0	19.7 ± 12.6	52.6 ± 18.8
Positive control—benzyl salicylate	444 ± 46.5	2154 ± 105	3198 ± 293

Values are a mean ± SD of triplicates detected in the medium after different timepoints

### Substances metabolised to salicylic acid

**Fig. 3:**
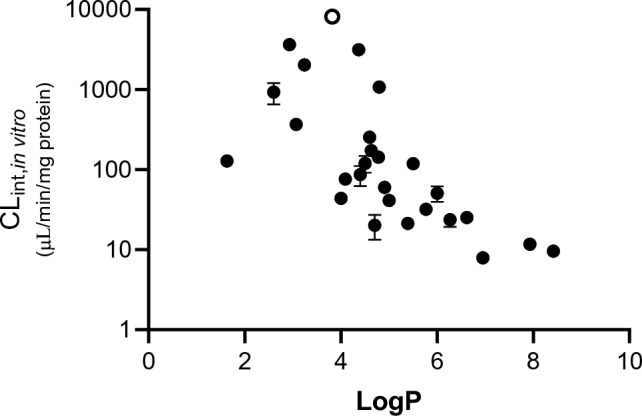
Intrinsic clearance (CL_int, in vitro_) of 27 salicylate esters measured in human liver S9. Values are a mean ± SD of n = 3. Phenyl salicylate is denoted with an open symbol

For the remaining 27 substances (all chemically defined as salicylate esters), the CL_int, in vitro_ values tended to increase with decreasing LogP (which ranged from 1.63 to 9.6), with the lowest value of 9.6 ± 0.3 µL/min/mg protein observed for the lipophilic long-chain salicylate, isotridecyl salicylate (LogP is 8.42), and much higher values for substances with a lower LogP, e.g., 3654.1 ± 192.6 µL/min/mg protein for acetaminosalol (LogP is 2.93) (Fig. 3). A notable outlier to this correlation was glycol salicylate, which has the lowest LogP of 1.63 but not the highest CL_int, in vitro_ value (the CL_int, in vitro_ value was only 128.0 ± 4.4 µL/min/mg protein). Another notable observation was that the highest CL_int, in vitro_ value of 8181.3 ± 680.6 µL/min/mg protein was for phenyl salicylate (denoted by an open symbol in Fig. 3), which has a mid-range LogP of 3.82.

### In vitro clearance assay reproducibility

**Fig. 4:**
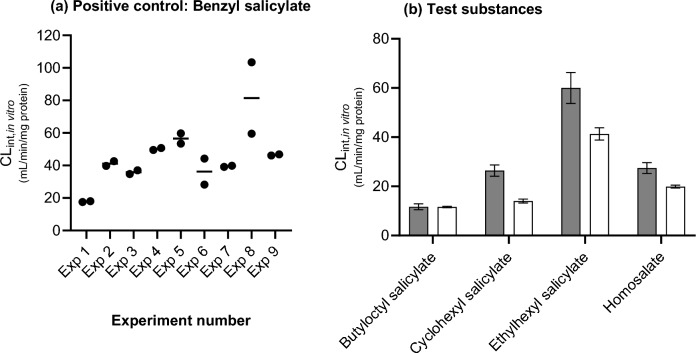
Reproducibility of the hydrolysis of A the positive control, benzyl salicylate, and B test substances, incubated in different experiments. Values are the intrinsic clearance (CL_int, in vitro_) mean ± SD, *n* = 3. The grey bars in B represent data from Experiment 1 and the white bars in B represent data from Experiment 9

As the test substances were incubated in batches of 5 per experiment, the reproducibility of the assay was assessed according to the intrinsic clearance values obtained for the positive control, benzyl salicylate, which was incubated in parallel in each experiment (and duplicate samples taken at 30 and 240 min only). Figure 4a shows that there was a good reproducibility within and between the assays, with the exception of experiment 8 in which there was a larger variation between duplicate samples and higher rate of hydrolysis of benzyl salicylate. The overall reproducibility within an experiment was very good for substances that were metabolised to salicylic acid, with a mean %CV of 9% (ranging from 0 to 30%).

Since the hydrolysis of the positive control run in Experiment 1 was lower than expected (based on the pilot study), all test substances run in this assay (butyloctyl salicylate, cyclohexyl salicylate, ethylhexyl salicylate, homosalate and isoamyl salicylate) were re-tested in a second experiment (Fig. 4b). This showed that despite the different rates of benzyl salicylate in the two experiments, the rate of hydrolysis of the test substances were either the same or slightly higher in the second experiment.

### HTPK modelling of the metabolism factor

Table 2 shows the comparison of the % of substance metabolised using in silico and/or in vitro inputs for seven esters of salicylic acid for which in vitro data for F_up_ and CL_int, in vitro_ values in primary human hepatocytes (PHH) were available from the Cosmetics Europe LRSS programme (unpublished). In addition, the drug, acetyl salicylic acid, was included in the evaluation since it was expected that 100% of this drug would be metabolised in 24 h based on human in vivo pharmacokinetics data for this drug (https://go.drugbank.com/drugs/DB00945; Needs and Brooks 1985). In vivo pharmacokinetics data were also available for homosalate and ethylhexyl salicylate (Matta et al. 2020). For these three substances with reported in vivo data, the % metabolised was predicted well by the HTPK Model using in silico only and/or measured in vitro F_up_ and PHH CL_int, in vitro_ data. When liver S9 CL_int, in vitro_ values (in 4% BSA) were used instead of PHH CL_int, in vitro_ values, the predicted % of homosalate and ethylhexyl salicylate metabolised was increased to ~ 70%. The combination of in silico *F*_up_ and liver S9 CL_int, in vitro_ values resulted in the highest estimation of the % metabolism of most substances.

Of the substances without in vivo data, metabolism values for methyl salicylate were predicted to be ~ 100% regardless of whether in silico or in vitro data, alone or in combination, were used as input. The predicted % of benzyl salicylate and isoamyl salicylate metabolised was lowest when in silico-only input were used. By contrast, when in vitro data for F_up_ and PHH or liver S9 CL_int, in vitro_ were used for these two substances they were predicted to be extensively metabolised (80–99% metabolised in 24 h). The predicted % of cyclohexyl salicylate metabolised in 24 h was also lowest when in silico-only input were used but there was a difference in the values depending on whether CL_int, in vitro_ values from PHH or liver S9 were used, with the latter resulting in a higher predicted % metabolism. In contrast to the other substances listed in Table 3, the predicted % metabolism of butyloctyl salicylate was lowest when CL_int, in vitro_ from either PHH or liver S9 were used in combination with in vitro *F*_up_. Values were higher (and similar) when an in silico value for *F*_up_ was used in combination with liver S9 or in silico CL_int_ values.

**Table 3:** Percentage of substance metabolised in 24 h: impact of in silico and measured values on the predicted metabolism factor

Substance	In vivo	In silico F_up_ + in silico CL_int_ %	In vitro F_up_^1)^ + CL_int, in vitro_ using PHH^2)^ %	In vitro F_up_^1)^ + CL_int, in vitro_ using S9 in 4% BSA^3)^ %	In silico F_up_ + CL_int, in vitro_ using S9 in 4% BSA^3)^ %
Acetyl salicylic acid	100% ^4)^	100.0%	Not determined	Not determined	Not determined
Homosalate	43.7% ^5)^ 19–30% ^6)^	42.6	45.7	67.8	80.8
Ethylhexyl salicylate	19.41–45.63% ^6)^	31.2	47.1	72.3	82.0
Methyl salicylate	Unknown	98.9	100.0	100.0	100.0
Benzyl salicylate	Unknown	42.2	79.8	98.8	96.7
Isoamyl salicylate	Unknown	31.9	93.1	92.6	93.6
Cyclohexyl salicylate	Unknown	29.3	63.0	80.1	91.1
Butyloctyl salicylate	Unknown	58.7	20.5	16.5	65.7

(1) In vitro F_up_ unpublished data were from the Cosmetics Europe LRSS programme. (2) Values for the in vitro CL_int_ in PHH (measured according to parent substance depletion, thus including multiple metabolite formation, were from the Cosmetics Europe LRSS Programme Boettcher et al. 2023; Grégoire et al. 2023) see values in Supplementary Table 3). (3) The correction for *F*_up_ is already included in the in vitro assay since 4% BSA was added. (4) Value was from human clinical data (https://go.drugbank.com/drugs/DB00945; Needs and Brooks 1985). (5) Value was calculated using allometric scaling from a third-party rat in vivo study in which the in vivo phase was conducted prior to the testing ban on March 11th, 2013 (SCCS 2020; Kim et al. 2014). (6) Value was from human clinical data (Matta et al. 2020)

**Fig. 5:**
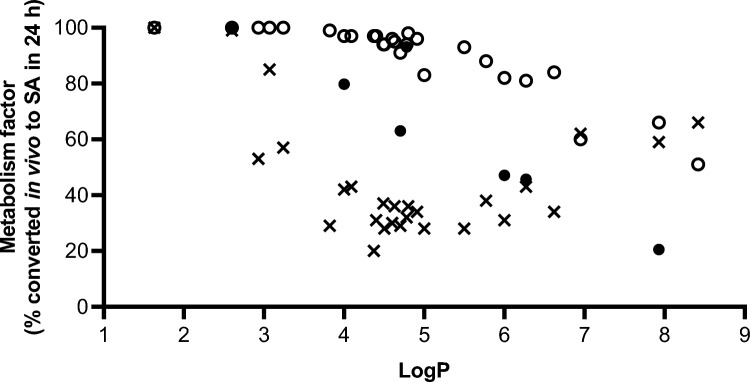
Predicted metabolism factors (expressed as a % of the substance metabolised in vivo in 24 h) for all substances metabolised by human liver S9. HTPK model predictions were using either (1) in silico values for the fraction unbound in plasma (*F*_up_) and intrinsic clearance (CL_int_) only (denoted by crosses) or (2) using in silico values for *F*_up_ and measured CL_int_ in liver S9 (denoted by open circles) or, (3) for the chemicals listed in Table 2 with measured CL_int_ in PHH and in vitro values for *F*_up_ (denoted by closed symbols)

The predicted metabolism factors for all esters of salicylic acid plotted against their LogP values are shown in Fig. 5. Measured values for F_up_ were not available for most of the substances; therefore, values shown are derived using in silico-only input and in silico *F*_up_ combined with measured CL_int, in vitro_ using human liver S9 (or PHH for 7 substances). The predicted metabolism factors derived from in silico F_up_ combined with liver S9 CL_int, in vitro_ values were an average of 2.4-fold higher than those when in silico-only input values were used. The predicted metabolism factors for 7 substances with CL_int, in vitro_ values from PHH (shown in Table 2) are also shown in Fig. 5. When PHH derived values were used as input, the resulting metabolism factors were generally higher than in silico-only predictions and lower than liver S9 derived predictions. There was a general trend of a lower metabolism factor with increasing LogP regardless of the source of CL_int,_ input.

## Discussion

This study aimed to derive a metabolism factor for individual salicylate esters to correct the SED as part of the probabilistic exposure estimate of salicylic acid after topical exposure to different salicylate esters present in cosmetic products. The metabolism factor represents the percentage of substance metabolised to salicylic acid in 24 h in vivo.

The in vitro study was conducted in two phases, the first to establish optimal incubation conditions and the second to measure the rate of substance hydrolysis using these optimal conditions. There were several considerations which impacted the design of the incubations. Normally, CL_int, in vitro_ values are derived from the slope of the depletion of the parent substance; however, in the current study, values were calculated based on the measurement of salicylic acid formation. The amount of parent substance was indirectly calculated by subtracting the concentration of salicylic acid formed from the initial nominal concentration. This is because each salicylate ester would have required individual analytical development and, together with the long run time of the HPLC analysis, this meant that the time needed to analyse the parent substance in each sample was much longer than that for the metabolite. By excluding the analysis of the salicylate esters and measuring salicylic acid only, the length of the main study was reduced from ~ 5 months to ~ 5 weeks. An additional reason for monitoring the metabolite generation, i.e. salicylic acid, was that the focus was on the rate of conversion to salicylic acid rather than the clearance of the parent substance or metabolites from other biotransformation pathways, e.g., glycine conjugation, sulfation and glucuronidation. In incubations with 0% BSA, 90–100% of the nominal concentrations of benzyl salicylate and methyl salicylate were converted to salicylic acid, indicating that loss of parent substance was indeed directly proportional to the formation of salicylic acid. This observation supports the use of this method for estimating hydrolysis and the associated metabolism values for these substances.

Human liver S9 was used in the incubations since this allowed for potential follow-up experiments to be performed in skin S9 so that a direct comparison of liver and skin metabolism could be made under same conditions (although this was not required since hydrolysis was already extensive using liver S9). The use of liver S9 also enabled the focus to be on Phase 1 metabolism. The incubations included NAD and NADPH for potential CYP-mediated metabolism to salicylic acid, but the main pathway of interest was hydrolysis via carboxylesterases. If PHH had been used, unknown amounts of metabolites other than salicylic acid could have been formed (as was observed in LRSS studies for 5 of the salicylate esters tested here Boettcher et al. 2023; Grégoire et al. 2023) and, as such, this would not have allowed the assumption that the formation of salicylic acid was directly proportional to the depletion of the parent substance. Indeed, the values for CL_int_ for PHH used to calculate the metabolism factor in Table 2 were derived using parent depletion and not salicylic acid formation, resulting in an overestimation of the conversion of the substances to salicylic acid.

In the pilot experiment, a concentration of 0.1 mg/ml S9 was tested instead of 2 mg/ml in previous studies conducted by others, e.g., Eilstein et al. (2020) and Lester et al. (2021). This is because hydrolysis of these esters is known to be rapid; therefore, if a higher S9 concentration had been used, there would have been a risk that the first timepoint would already have indicated that 100% of the parent had been converted to salicylic acid, making the calculation of an intrinsic clearance value less accurate. The lower S9 concentration was expected to result in a slower formation of salicylic acid, which could be captured in at least 3 timepoints (thus increasing the confidence in the associated intrinsic clearance value). The pilot experiment data showed a steady formation of salicylic acid from benzyl and methyl salicylate under the conditions used. The rate of production was captured over the first 4 timepoints, indicating that the concentration of human liver S9 used resulted in a suitable rate of conversion for esters of salicylic acid which were suspected of being rapidly metabolised.

Many of the substances under investigation have a high LogP value and are thus likely to exhibit high, non-specific binding to plastic vessels. Therefore, glass-coated plates were used with the aim to reduce this effect. These data demonstrated low or no non-specific binding to the glass-coated vessel walls, with little binding of benzyl salicylate or methyl salicylate in incubations without BSA. Non-specific binding can also be reduced by adding protein, e.g., BSA to the incubation; therefore, the pilot study also investigated the impact of adding 1% and 4% BSA to the medium. The initial rate of salicylic acid production from methyl salicylate was unaltered by the presence of increased concentration of BSA. This indicates that the affinity of this substance for plasma proteins was low such that it was readily released from the protein, thus making it available for metabolism. The rate of salicylic acid formation from benzyl salicylate was decreased with increasing BSA concentrations, indicating that the free fraction of parent substance available for metabolism was lower in the presence of BSA. However, when the t_1/2_ values from the incubations were scaled to in vivo hepatic clearance (CL_H_), the values were the same regardless of the concentration of BSA. This means that the design of the main experiments included 4% BSA to represent in vivo plasma binding, reduce non-specific binding and increase the solubility of the more lipophilic substances. Human plasma contains approximately 4% (40 g/L) serum albumin (Haab et al. 2005), hence the addition of 4% (bovine) serum albumin to the media made the in vitro environment with regards to protein binding and free/biological available fraction of the substance more similar to physiological conditions.

The number of substances tested in a single experiment was no more than five to be able to analyse all the samples directly after they were generated. The same batch of liver S9 was used for all experiments and the samples generated were analysed immediately after generation to prevent any degradation or hydrolysis during storage. These precautions were expected to decrease the likelihood of inter-experimental variation due to inter-batch variability or sample storage and freeze–thaw processes. Indeed, the intra- and inter-experimental reproducibility of these assays was very good, thereby allowing a comparison of the CL_int, in vitro_ values between the substances to be made.

There was a general trend of higher CL_int, in vitro_ values correlating with decreasing LogP values. This correlation is in accordance with the substrate preference of the main liver carboxylesterase enzyme (carboxylesterase-1) responsible for the hydrolysis of ester-containing substrates (Fagerberg et al. 2014; Ross et al. 2012), which preferentially hydrolyses esters with a large, bulky acyl group and a small alcohol group. This correlation with the LogP was demonstrated previously with a panel of 16 paraben esters, whereby short-chain parabens with a low LogP were more rapidly metabolised by liver S9 than long-chain lipophilic parabens (Lester et al. 2021). Another observation made by Lester et al. (2021) was that parabens with a phenyl or benzyl side group resulted in much higher CL_int, in vitro_ values compared to straight-chain parabens. This was also observed in the current study, whereby phenyl salicylate exhibited the highest CL_int, in vitro_ value of all the substances tested, despite its mid-range LogP value. The presence of the phenyl group on esters appears to make them very good carboxylesterase substrates. By contrast, glycol salicylate appears to be a poorer carboxylesterase substrate than its low LogP would indicate.

There were several substances tested in human liver S9 incubations which were not metabolised to salicylic acid at all (methyl methyl salicylate, potassium methoxysalicylate and silandiol salicylate) or only trace amounts were formed (chlorosalicylic acid). While these might appear to be substances that may lead to salicylic acid exposure due to their nomenclature, they are not chemically defined as esters of salicylic acid and, as such, were not expected to produce salicylic acid in significant amounts, which was confirmed in the current study. The trace amounts of salicylic acid formed from chlorosalicylic acid is unlikely to be due to hydrolysis because it would mean the loss of a chloride ion, rather than cleavage of the ester bond (dechlorination could occur via CYPs, since NADPH and NADH were added to the incubation). For this substance, the formation of salicylic acid in vivo is predicted to be extremely slow and the contribution to the aggregate exposure negligible. This finding suggests that the nomenclature or chemical similarity of substances could be a starting point for grouping them according to their potential to contribute to salicylic acid exposure; however, additional considerations should be incorporated, namely, a metabolic pathway-based category, whereby grouping is with respect to metabolism to a common metabolite (in this case, salicylic acid). We have shown that in vitro metabolic studies (e.g., using liver S9) can be used to help identify metabolic pathways and detect the formation of salicylic acid. This study highlights the importance of conducting in vitro experiments to determine the quantitative contribution of substances to salicylic acid exposure.

The model selected to convert CL_int, in vitro_ values for the metabolism to salicylic acid into in vivo metabolism factors was the HTPK Model from SimulationPlus, which uses simple equations to derive in vivo half-lives. This simulates the disposition of a chemical with a single central compartment instead of the whole-body PBPK incorporated into more complex full PBPK models (Naga et al. 2022). This was preferred over more complex PBPK models to help with transparency, while still providing a good prediction of in vivo metabolism kinetics. Indeed, Naga et al. (2022) demonstrated that results from the HTPK and a full PBPK modelling approach were comparable but that the HTPK model reduced the simulation time from hours to seconds.

While in silico only-based predictions for acetylsalicylic acid, homosalate and ethylhexyl salicylate correlated well with their in vivo metabolism, it cannot be assumed that the in silico only-based HTPK model predicts the metabolism of all substances with equal accuracy, especially since these tended to result in lower metabolism factors than when in vitro values for F_up_ and liver S9 CL_int, in vitro_ were used as input. Indeed, there was a marked difference in the predicted rate of metabolism of benzyl salicylate, isoamyl salicylate, cyclohexyl salicylate and butyloctyl salicylate between in silico-only and in vitro-only input. Although it is not known which value correlates best with their in vivo clearance, this finding suggests that for some substances, the use of in vitro data as inputs may result in the more conservative value for the metabolism factor compared to those derived from only in silico predictions. The combination of in silico F_up_ and liver S9 CL_int, in vitro_ values resulted in the highest metabolism factors for most substances, indicating that (a) measured values of F_up_ for some of these may be required to refine the prediction further and (b) liver S9 CL_int, in vitro_ values provide a more conservative metabolism factor than PHH derived CL_int, in vitro_ values. Notably, S9 fractions do not have a plasma membrane; therefore, uptake into cells is not considered in the measurement of the metabolism (which could be a limiting factor for metabolism for several of the salicylate esters). Values for homosalate and ethylhexyl salicylate were higher when liver S9 values were used instead of PHH, indicating that the design of the liver S9 assay favoured metabolism (due to a lack of plasma membrane) compared to PHH. Further investigations using hepatocytes to generate intrinsic clearance values according to salicylate formation and parent ester depletion could be conducted to refine the predictions of metabolism. Future studies could also include more complex PBPK models incorporating a skin compartment to predict the internal exposure to salicylic acid for each substance after topical application; however, the current work focused on the generation of metabolism factors which were separate from skin absorption values.

In conclusion, to help with the estimation of the exposure to salicylic acid from the use of salicylate esters in cosmetic products, we propose an additional correction factor by which their potential contribution to the aggregate exposure can be evaluated. This adjustment factor accounts for their rate of metabolism to salicylic acid, which is used in conjunction with an adjustment factor relating to their skin absorption and the conversion of the MW of the parent substance to the equivalent amount of salicylic acid. This proposed strategy is specific to salicylate esters. An analysis would be needed to verify whether a similar methodology can be applied for future evaluations requiring the assessment of aggregate exposure to a common metabolite.

### Supplementary Information

Below is the link to the electronic supplementary material.

Supplementary file1 (XLSX 153 KB)

Supplementary file2 (XLSX 72 KB)

Supplementary file3 (DOCX 14 KB)

Supplementary file4 (DOCX 15 KB)

## Data Availability

Data are available upon request to the corresponding author, Karim Najjar.

## Abbreviations

Q_H_: Blood flow to the liver
R_bp_: Blood-to-plasma ratio
BW: Body weight
BSA: Bovine serum albumin
F_up_: Fraction unbound in the plasma
t_1/2_: Half-life
HTPK: High-throughput pharmacokinetic
INCI: International Nomenclature of Cosmetic Ingredient
CL_int, in vitro_: In vitro intrinsic clearance
IVIVE: In vitro to in vivo extrapolation
LC–HRMS: Liquid chromatography–high resolution mass spectrometry
LC–MS: Liquid chromatography–mass spectrometry
LOQ: Limit of quantification
LRSS: Long range science strategy
PHH: Primary human hepatocytes
CL_int_: Restricted CL_int_ values
SCCS: Scientific Committee on Consumer Safety
SED: Systemic exposure dose
ISTD: Internal standard
CL_H_: In vivo hepatic clearance
V_d_: Volume of distribution in steady state
