# Supplementary material for: In vitro to in vivo extrapolation to derive a metabolism factor for estimating the aggregate exposure to salicylic acid after dermal exposure of its esters
Source: Arch Toxicol. 2024 Apr 24;98(7):2199–211. doi: 10.1007/s00204-024-03749-8 (PMC11169020; doi:10.1007/s00204-024-03749-8)
Supplement: Supplementary file 3 — Supplementary file3 (DOCX 14 KB) [file 204_2024_3749_MOESM3_ESM.docx]

Supplementary Table 2: HPLC gradients (LC-MS/MS analysis)

| **Salicylic acid (Q-Exactive/Q-Exactive Plus)** | | | | | | | |
| --- | --- | --- | --- | --- | --- | --- | --- |
| [min] | | 0.0 | 0.1 | 0.6 | 2.8 | 2.9 | 5.0 |
| Mobile phase | Acetonitrile + 0.1% formic acid | 5 | 5 | 97 | 97 | 5 | 5 |
|  | H_2_O + 0.1% formic acid | 95 | 95 | 3 | 3 | 95 | 95 |
| **7-ethoxycoumarin (Triple quadrupole)** | | | | | | | |
| [min] | | 0 | 0.1 | 0.8 | 1.7 | 1.8 | 2.5 |
| Mobile phase | Acetonitrile + 0.1% formic acid | 5 | 5 | 97 | 97 | 5 | 5 |
|  | H_2_O + 0.1% formic acid | 95 | 95 | 3 | 3 | 95 | 95 |
