# Supplementary material for: In vitro to in vivo extrapolation to derive a metabolism factor for estimating the aggregate exposure to salicylic acid after dermal exposure of its esters
Source: Arch Toxicol. 2024 Apr 24;98(7):2199–211. doi: 10.1007/s00204-024-03749-8 (PMC11169020; doi:10.1007/s00204-024-03749-8)
Supplement: Supplementary file 4 — Supplementary file4 (DOCX 15 KB) [file 204_2024_3749_MOESM4_ESM.docx]

Supplementary Table 3. MS and chromatographic parameters (LC-MS/MS)

| **Orbitrap^TM^ (Q-Exactive, Q-Exactive Plus)** | | | | | | | | | | |
| --- | --- | --- | --- | --- | --- | --- | --- | --- | --- | --- |
| Compound | Molecular weight | | Exact mass [M-H]^-^ (m/z) | | Scan range (m/z) | | Mass resolution | | Retention time (min) | |
| Salicylic acid | 138.12 | | 137.023 | | 120 – 900 or  100 – 900 | | 70,000 | | 2.15 | |
| Warfarin (ISTD) | 308.33 | | 307.099 | | 120 – 900 | | 70,000 | | 2.41 | |
| Diclofenac (ISTD) | 296.15 | | 294.008 | | 100 – 900 | | 70,000 | | 2.49 | |
| **Triple quadrupole** | | | | | | | | | | |
| Compound | | Molecular weight | [M+H]^+^  (m/z) | Monitoring ion (m/z) | | Scan time (s) | | Collision energy (V) | | Retention time  (min) |
| 7-ethoxycoumarin | | 190.20 | 191.0 | 163.0 | | 0.010 | | 20 | | 1.29 |
| Diazepam (ISTD) | | 284.74 | 285.0 | 193.0 | | 0.010 | | 35 | | 1.32 |
